# Intra- and inter-hemispheric effective connectivity in the human somatosensory cortex during pressure stimulation

**DOI:** 10.1186/1471-2202-15-43

**Published:** 2014-03-21

**Authors:** Yoon Gi Chung, Sang Woo Han, Hyung-Sik Kim, Soon-Cheol Chung, Jang-Yeon Park, Christian Wallraven, Sung-Phil Kim

**Affiliations:** 1 Department of Brain and Cognitive Engineering, Korea University, Seoul, Republic of Korea; 2 Department of Biomedical Engineering, Research Institute of Biomedical Engineering, College of Biomedical & Health Science, Konkuk University, Chungju, Republic of Korea; 3 School of Design and Human Engineering, Ulsan National Institute of Science and Technology, Ulsan, Republic of Korea

## Abstract

**Background:**

Slow-adapting type I (SA-I) afferents deliver sensory signals to the somatosensory cortex during low-frequency (or static) mechanical stimulation. It has been reported that the somatosensory projection from SA-I afferents is effective and reliable for object grasping and manipulation. Despite a large number of neuroimaging studies on cortical activation responding to tactile stimuli mediated by SA-I afferents, how sensory information of such tactile stimuli flows over the somatosensory cortex remains poorly understood. In this study, we investigated tactile information processing of pressure stimuli between the primary (SI) and secondary (SII) somatosensory cortices by measuring effective connectivity using dynamic causal modeling (DCM). We applied pressure stimuli for 3 s to the right index fingertip of healthy participants and acquired functional magnetic resonance imaging (fMRI) data using a 3T MRI system.

**Results:**

DCM analysis revealed intra-hemispheric effective connectivity between the contralateral SI (cSI) and SII (cSII) characterized by both parallel (signal inputs to both cSI and cSII) and serial (signal transmission from cSI to cSII) pathways during pressure stimulation. DCM analysis also revealed inter-hemispheric effective connectivity among cSI, cSII, and the ipsilateral SII (iSII) characterized by serial (from cSI to cSII) and SII-level (from cSII to iSII) pathways during pressure stimulation.

**Conclusions:**

Our results support a hierarchical somatosensory network that underlies processing of low-frequency tactile information. The network consists of parallel inputs to both cSI and cSII (intra-hemispheric), followed by serial pathways from cSI to cSII (intra-hemispheric) and from cSII to iSII (inter-hemispheric). Importantly, our results suggest that both serial and parallel processing take place in tactile information processing of static mechanical stimuli as well as highlighting the contribution of callosal transfer to bilateral neuronal interactions in SII.

## Background

Four types of mechanosensitive afferents mediate the sense of touch in the human skin including slow-adapting type I (SA-I) afferents for low-frequency (static) stimuli, slow-adapting type II (SA-II) afferents for skin stretching, fast-adapting type I (FA-I) afferents for flutter, and fast-adapting type II (FA-II) afferents for high-frequency (vibratory) stimuli [1,2]. During mechanical stimulation, they project sensory signals to the somatosensory cortical regions for tactile perception [3]. Among them, the somatosensory projection from SA-I afferents is the most effective and reliable for object grasping and manipulation [4-6] with its characteristic spatial responses [7-9].

It is of great interest to understand how tactile information is processed over somatosensory cortical networks. Hierarchical organization of tactile information processing in the primary (SI) and secondary (SII) somatosensory cortices has been documented in many anatomical [10-12] and neuroimaging studies [13-17]. However, there is ongoing debate concerning whether tactile information is processed in serial (relay of sensory signals from SI to SII) or in parallel (relay of sensory signals to both SI and SII). Recently, dynamic causal modeling (DCM) with functional magnetic resonance imaging (fMRI) data has been proposed to address this issue. DCM treats the brain as a dynamic system to identify effective connectivity among brain regions, i.e. which regions cause activity in target regions based on model estimation and regional coupling parameters. In DCM, serial processing models for somatosensory cortical networks hypothesize sequential transduction of sensory inputs from SI to SII [15], whereas parallel processing models hypothesize bifurcated transduction of sensory inputs to both SI and SII [16]. A recent fMRI study by Liang et al. suggested that parallel processing models may better explain effective connectivity in SI and SII for electrical and thermal stimuli [16]. In contrast, another fMRI study by Kalberlah et al. suggested that serial processing models may better explain effective connectivity in SI and SII for vibrotactile stimuli [15]. Thus, these reports suggest that a hypothesis for sensory signal transduction between SI and SII should be evaluated depending upon the type of tactile stimuli. In this study, we focused on tactile information processing models for static (e.g. pressure) stimuli. In addition to previous studies that only examined *intra-hemispheric* networks, we further aimed to investigate *inter-hemispheric* networks across SI and the bilateral SII. To our knowledge, no human fMRI study has investigated effective connectivity *across* hemispheres for any mechanical stimulus.

In the present study, we addressed two questions regarding somatosensory networks associated with SA-I afferents: first, we addressed how intra-hemispheric effective connectivity is formed in the contralateral SI (cSI) and SII (cSII) for tactile information processing of pressure stimuli. We employed DCM to clarify whether pressure stimuli are processed in serial or in parallel with similar hypotheses to previous reports [15,16]: (1) the serial processing model hypothesis highlighting sequential inputs from cSI to cSII and (2) the parallel processing model hypothesis highlighting two-way inputs to cSI and cSII. Second, we addressed how inter-hemispheric effective connectivity is formed across cSI, cSII, and the ipsilateral SII (iSII) for tactile information processing of pressure stimuli. Consequently, we performed a second DCM analysis to assess three possible models: (1) the first model hypothesized causal activity (an information flow) from cSI to iSII; (2) the second model hypothesized causal activity from cSII to iSII; and (3) the third model hypothesized causal activity from cSI to iSII as well as from cSII to iSII. Using the human fMRI data recorded from our pressure stimulation experiment, we evaluated each hypothesis to find which sensory signal transduction model most likely explained the neural data.

## Methods

### Participants

Twenty-one healthy volunteers (age, 24.19 ± 2.71 years; all right-handed) with no history of neurological disorders or deficits in tactile processing gave written informed consent and participated in this study, which was approved by the Korea University Institutional Review Board (KU-IRB-11-46-A-1).

### Pressure stimulation

**Figure 1:**
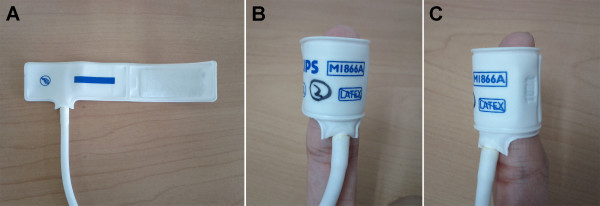
**An MR compatible stimulation device.** A neonatal cuff **(A)** wrapped around the right index fingertip (**B**: front view; **C**: side view) exerted a pressure stimulus of 5.98 g/mm^2^ by expanding at the turn-on of the pump in a blood pressure monitor through an elastic air-tube. It pressed the whole ventral surface of the fingertip for 3 s in each stimulus period.

A band-type MR-compatible stimulation device built by our group (Figure 1) [18] was used to apply a pressure stimulus of 5.98 g/mm^2^ to the right index fingertip. A neonatal cuff (M1866A, Philips Healthcare, Best, The Netherlands) wrapped around the fingertip was directly connected to a rolling pump in a blood pressure monitor (BP3AG1, Microlife AG, Widnau, Switzerland) through an elastic air-tube with a length of 5 m and a diameter of 4 mm. The cuff was controlled by a pressure sensor for achieving uniform pressure; the sensor in turn was controlled by E-Prime 2.0 software (Psychology Software Tools, Inc., Sharpsburg, PA, USA) for configuring the length of stimulation. The cuff expanded at the turn-on of the pump and pressed the whole ventral surface of the fingertip (see Kim et al. for further information concerning the stimulation device [18]).

Participants were instructed to lie comfortably on the MRI table with eyes closed during the scanning in each session, to put earplugs in their ears, and to hold an emergency squeeze-ball in their left hands during the entire scanning session. Before scanning, the cuff was attached to wrap participants' right index fingertips with minimal pressure on the skin. Each participant performed four block-designed study sessions. Each session consisted of four trials. To avoid potential adaptation due to repetitive stimulus application, we designed four separate sessions instead of one session, totaling 16 trials. A single trial comprised a 21 s resting period followed by a 3 s stimulation period. During each stimulation period, a single static indentation was applied continuously to the participant's right index fingertip.

### Anatomical and functional data acquisition

Anatomical and functional images were acquired using a 3T MRI system (Magnetom TrioTim, Siemens Medical Systems, Erlangen, Germany) with a standard 32-channel head coil. T_1_-weighted anatomical images were acquired using a 3D magnetization-prepared gradient echo (MPRAGE) sequence, with the imaging parameters of repetition time (TR) = 1,900 ms, echo time (TE) = 2.48 ms, flip angle = 9°, field of view (FOV) = 200 mm, and voxel size = 0.8 × 0.8 × 1 mm^3^. $T_{2}^{*}$-weighted functional images were acquired using a gradient echo-planar imaging (EPI) sequence, with the imaging parameters of TR = 3,000 ms, TE = 30 ms, flip angle = 90°, FOV = 240 mm, slice thickness = 3 mm, and voxel size = 3 × 3 × 3 mm^3^.

### Statistical analysis

Functional images were preprocessed using SPM8 (Wellcome Department of Imaging Neuroscience, UCL, London, UK), through a series of steps of slice-timing correction, realignment with the rigid-body transformation matrices, normalization to the Montreal Neurological Institute (MNI) template, and smoothing with an 8 mm full-width-half-maximum (FWHM) isotropic Gaussian kernel. The mean EPI image of each individual subject was directly warped into the standard EPI template in SPM8 during the normalization step. Then, the conventional general linear model (GLM) in SPM8 performed statistical analyses on blood oxygenation level-dependent (BOLD) signals with a canonical hemodynamic response function and its time and dispersion derivatives. A 128 s high-pass filter removed physiological artifacts in the BOLD signals. A full factorial design based on a random effects model performed a group analysis for the inference of statistically significant cortical activation. Cluster-level *F*-statistics (*p* < 0.05 with a family-wise-error (FWE) correction, with a minimum threshold (*k*) of 5 voxels for significant clusters) produced group-level statistical parametric maps (SPMs) representing significant voxel clusters. The automated anatomical labeling toolbox [19] determined anatomical cluster labels of the activation regions in the SPMs.

### DCM analysis

**Figure 2:**
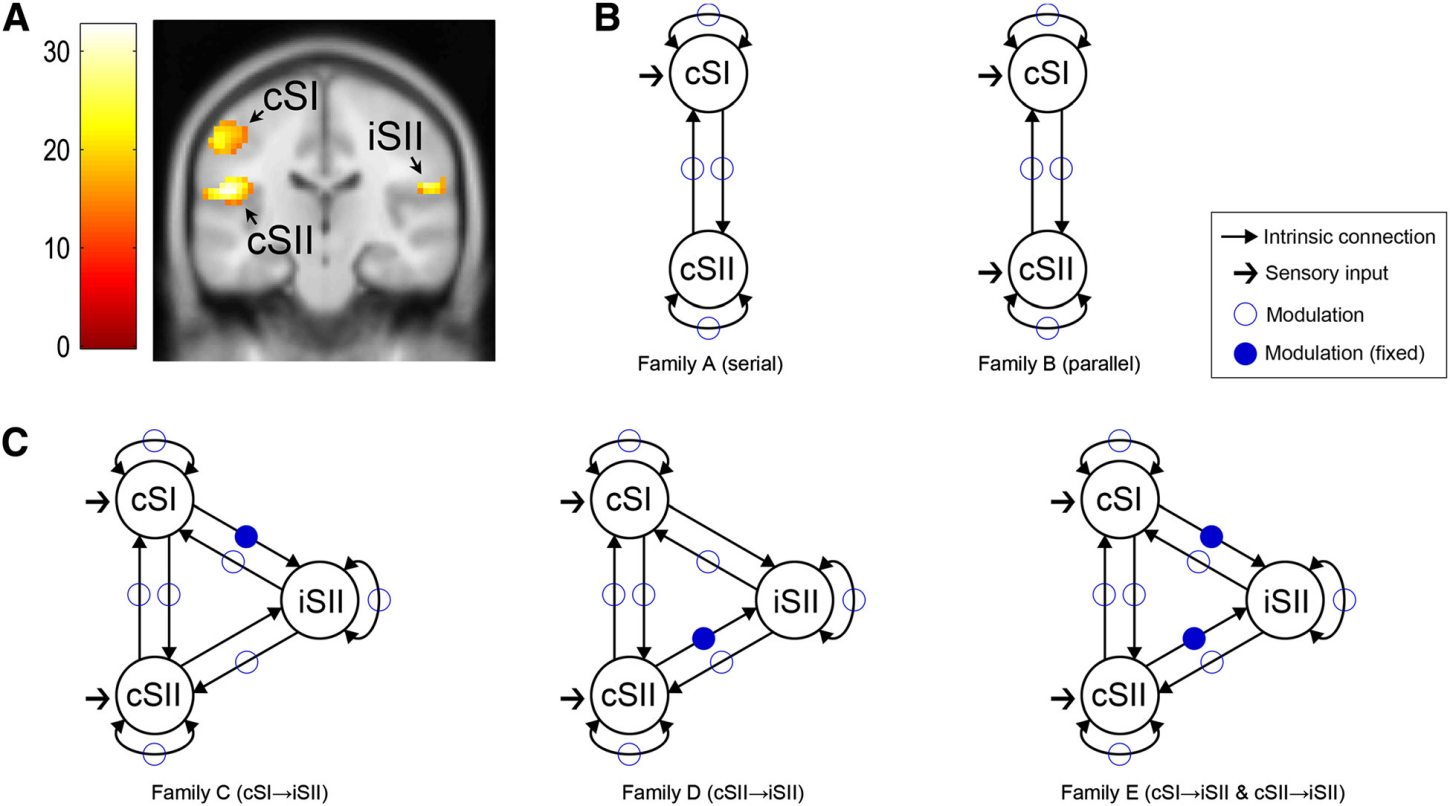
**ROIs and model families for DCM analysis. (A)** Three seed ROIs are shown in cSI, cSII, and iSII (group analysis with 21 participants, *F*-test, *p*_(FWE)_ < 0.05, *k* > 5, bar: *F*-statistics) **(B)** Two intra-hemispheric model families were constructed to find out whether pressure stimuli were processed in serial (left: family A) or parallel (right: family B) in cSI and cSII. With full intrinsic connectivity (thin arrows), we defined at least one and at most four modulations (empty circles) in the intrinsic connections. A key difference between family A and family B was that one driving input (a thick arrow) was applied to only cSI in family A, whereas two driving inputs (two thick arrows) were applied to both cSI and cSII. **(C)** Three inter-hemispheric model families were constructed to find out how pressure stimuli were delivered within cSI, cSII, and iSII: from cSI to iSII (left), from cSII to iSII (middle), or both (right). With full intrinsic connectivity, we defined a fixed modulation (filled circles) from cSI to iSII but no modulation from cSII to iSII (left), a fixed modulation from cSII to iSII but no modulation from cSI to iSII (middle), and two fixed modulation from cSI to iSII and from cSII to iSII (right). At least one modulation (empty circles) was applied to the intrinsic connections. Two driving inputs (two thick arrows) were applied to both cSI and cSII.

We used the Anatomy toolbox [20] to generate anatomical masks for three seed regions of interests (ROIs), including cSI (Brodmann area (BA) 3a, 3b, 1, and 2) [21,22], cSII, and iSII (parietal operculum (OP) 1, 2, 3, and 4) [23,24] for DCM analysis (Figure 2A). DCM infers effective connectivity by estimating parameters of regional coupling using a Bayesian framework in dynamic systems of neuronal populations, which are unobservable directly from BOLD signals. DCM is a hypothesis-driven approach, and finds optimal model parameters at the neuronal level to make BOLD signals generated with predefined hypotheses as close to observed BOLD signals as possible. We used a bilinear state equation with three components: (1) experimental (driving) inputs perturbing brain states, i.e., in our case, sensory signals directly projecting to the cortex; (2) intrinsic connectivity in the absence of experimental perturbations; and (3) changes (modulations) of the intrinsic connectivity induced by experimentally manipulated inputs, i.e. changes in regional couplings by sensory inputs, which provided information concerning how much activation in source regions receiving direct inputs caused an increase/decrease in activation in target regions per unit of time [25]. The resulting modulations were the components of interest in this study and were used to model the flow of tactile information among the somatosensory cortical regions. Bayesian model selection (BMS) in DCM was achieved by a free energy approximation to the log evidence of each model in terms of model fit and complexity. BMS determined which model was the most preferred among defined models [26,27].

To model intra- and inter-hemispheric effective connectivity in this study, we used DCM instead of other methods for effective connectivity such as Granger causality [28] and structural equation modeling [29] because DCM has some advantages that (1) it provides direct modeling of effective connectivity between brain regions at the hidden neural level [25]; (2) it explains the relationship between neural activity and BOLD hemodynamic responses [25,26]; (3) it is less liable to be affected by the variability of hemodynamic response functions [30,31]; and (4) it provides modeling of effective connectivity caused by experimental perturbations [25,31].

Three seed ROIs were selected in each participant from individual analyses using the anatomical masks with cluster-level *F*-statistics (uncorrected *p* < 0.001, *k* > 5). Among 21 participants, six participants who showed no significant cluster for at least one of the three seed ROIs were excluded. Then, in each of the remaining 15 participants, we defined volumes of interests (VOIs) as spheres of 6 mm radius centered on the most significant peaks of individual ROIs. From the BOLD signals of all voxels in each VOI, the first eigenvariate was extracted as a representative time-series. Table 1 shows the average MNI coordinates and the average numbers of voxels across participants in cSI, cSII, and iSII.

**Table 1:** The average MNI coordinates and the average number of voxels in VOIs (15 participants)

**VOIs**	**Average MNI coordinates (mm)**			**Average number of voxels**
	**x**	**y**	**z**	
cSI	−51 (±1)	−20 (±2)	49 (±2)	25 (±2)
cSII	−50 (±2)	−15 (±1)	17 (±1)	27 (±1)
iSII	52 (±2)	−13 (±1)	16 (±1)	23 (±2)

cSI: contralateral primary somatosensory cortex; cSII: contralateral secondary somatosensory cortex; iSII: ipsilateral secondary somatosensory cortex; numbers in parentheses: standard error.

We performed two DCM analyses. First, we constructed two intra-hemispheric model families corresponding to serial processing (family A) or parallel processing (family B) between cSI and cSII. We defined full intrinsic connectivity due to the fact that both cSI and cSII were fully anatomically connected [32,33]. It led to four intrinsic connections: self-connections in each of cSI and cSII, a connection from cSI to cSII, and a connection from cSII to cSI. We presumed that at least one and at most four connections could be simultaneously modulated, as tactile sensory inputs would affect the dynamics of at least one intrinsic connection. This resulted in 15 (2^4^ - 1) models of modulations excluding a case with no modulation. Thus, there were a total of 30 models including 15 in family A and 15 in family B. A key difference between model families was that one driving input (a pressure stimulus) was applied only to cSI in family A, whereas two driving inputs were applied both to cSI and cSII in family B. In the present study, we examined a total of 450 models (combinations of 2 families, 15 models, and 15 participants) (Figure 2B).

Next, we constructed three inter-hemispheric model families to infer how the information of pressure stimuli was conveyed among cSI, cSII, and iSII. We again defined full intrinsic connectivity between all pairs of three VOIs due to the fact that cSI, cSII, and iSII were fully anatomically connected [34,35]. This resulted in 9 connections (3 self-connections and 6 bidirectional connections between three VOIs) with 512 (= 2^9^) modulation models. We applied two driving inputs to both cSI and cSII. To explain tactile information flows over inter-hemispheric connections, we built three model families. Each family consisted of a set of models with specific modulations being fixed: those fixed modulations represented pre-determined information pathways in accordance with our three hypotheses. We hypothesized that iSII received tactile information from cSI in family C (cSI → iSII), from cSII in family D (cSII → iSII), and from both cSI and cSII in family E (both cSI → iSII and cSII → iSII). Composition of modulation models in each family was determined as follows.

In family C, we first determined a fixed modulation in cSI → iSII and no modulation in cSII → iSII, which left 128 (= 2^7^) out of 512 models to be configured. Similarly, there were also 128 configurable models by determining a fixed modulation in cSII → iSII and no modulation in cSI → iSII in family D, or by determining fixed modulations in both cSI → iSII and cSII → iSII in family E. Then, among 128 models in each family, we excluded those models that had modulation in neither cSI → cSII nor cSI ← cSII as we avoided a case when there was no intra-hemispheric connectivity but inter-hemispheric connectivity. In other words, we considered integrating inter-hemispheric connectivity models with intra-hemispheric models by assuming that inter-hemispheric connectivity should involve intra-hemispheric connectivity. This exclusion procedure removed 32 models (= 2^5^) from 128, resulting in 96 models for each family. Thus, in our inter-hemispheric DCM analysis, we examined a total of 4320 models (combinations of 3 families, 96 models, and 15 participants) per session (Figure 2C).

To assess the fitness of each model to our experimental data, we performed BMS based on a random effect inference assuming that model structures could vary across participants [26,27]. BMS determined the best model and the best model family by computing exceedance probabilities of all models and model families. The exceedance probability inferred the probability that a specific model (or family) described the data better than any other model (or family) being compared [36]. Finally, the influence of modulation in the best models were evaluated using *t*-tests to ascertain whether modulatory parameters were statistically significant across participants with the null hypothesis being that differences were equal to zero.

## Results

**Figure 3:**
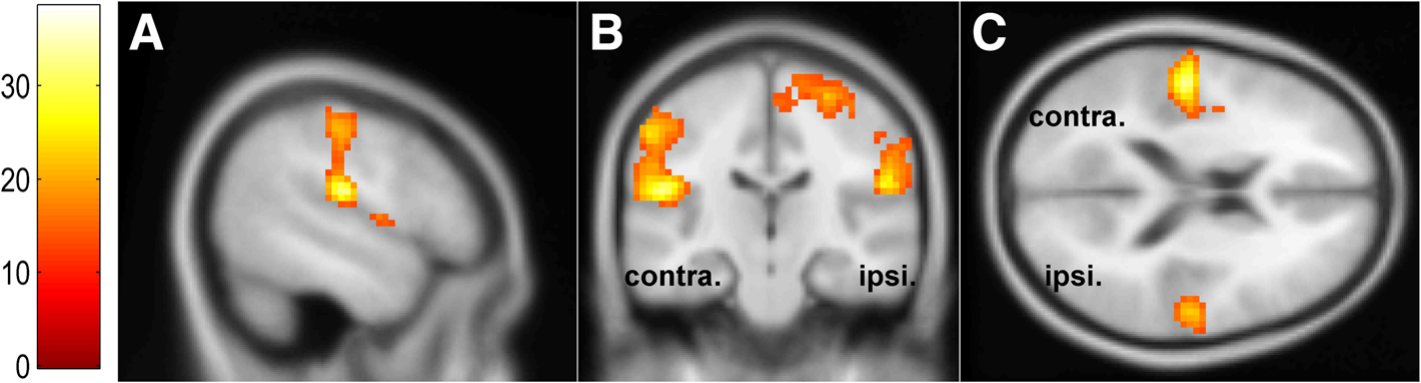
**Cortical activation during pressure stimulation.** GLM analysis revealed significantly activated clusters in the insula, postcentral gyrus, rolandic operculum, supramarginal gyrus, precentral gyrus, median cingulate, and paracentral lobule (group analysis with 21 participants, *F*-test, *p*_(FWE)_ < 0.05, *k* > 5, bar: *F*-statistics, **A**: sagittal view, **B**: coronal view, **C**: transverse view). The left side at B and the upper side at C indicate the contralateral hemisphere (contra.); the right side at B and the bottom side at C indicate the ipsilateral hemisphere (ipsi.); and activation at A indicates all suprathreshold voxels from both the contralateral and ipsilateral hemispheres.

As we used simple and distinct pressure stimuli with forces sufficiently higher than the absolute sensitivity threshold for the fingertips [37], all participants clearly felt the induced static indentation on their ventral surfaces of the right index fingertips for 3 s and confirmed this sensation in post-hoc interviews. Table 2 shows detailed information concerning significant clusters of activation during pressure stimulation (*F*-test, *p*_(FWE)_ < 0.05, *k* > 5). These results highlighted that cortical regions known to be related to tactile perception were activated in response to pressure stimulation during our experiment (Figure 3).

**Table 2:** **Significantly activated clusters during pressure stimulation (group analysis with 21 participants, *F*-test, *p***_**(FWE)**_ **< 0.05, *k*** **> 5)**

**Anatomical labels**		**MNI coordinates (mm)**			**Voxels**	***F***	***Z***	***p***_**(FWE)**_
		**x**	**y**	**z**				
**Insula**	**R**	**42**	**3**	**9**	**116**	**38.41**	**7.43**	**0.000**
Insula	R	39	−3	−3		23.81	6.20	0.000
**Postcentral gyrus**	**L**	**−51**	**−21**	**18**	**527**	**32.57**	**7.00**	**0.000**
Insula	L	−39	−3	9		30.26	6.81	0.000
Inferior parietal lobule	L	−57	−21	45		23.71	6.19	0.000
**Rolandic operculum**	**R**	**54**	**−21**	**21**	**220**	**26.79**	**6.50**	**0.000**
Postcentral gyrus	R	60	−18	33		19.38	5.68	0.000
Postcentral gyrus	R	51	−21	45		14.60	4.99	0.017
**Precentral gyrus**	**R**	**30**	**−24**	**60**	**207**	**23.12**	**6.13**	**0.000**
Precentral gyrus	R	24	−15	72		20.81	5.86	0.000
Paracentral lobule	R	12	−27	72		18.51	5.57	0.001
**Median cingulate**	**L**	**−6**	**9**	**36**	**6**	**16.85**	**5.34**	**0.003**
**Insula**	**R**	**33**	**27**	**3**	**22**	**16.61**	**5.30**	**0.004**
**Insula**	**L**	**−30**	**21**	**6**	**8**	**15.61**	**5.15**	**0.008**
**Paracentral lobule**	**L**	**−9**	**−27**	**66**	**11**	**14.52**	**4.98**	**0.018**

**Figure 4:**
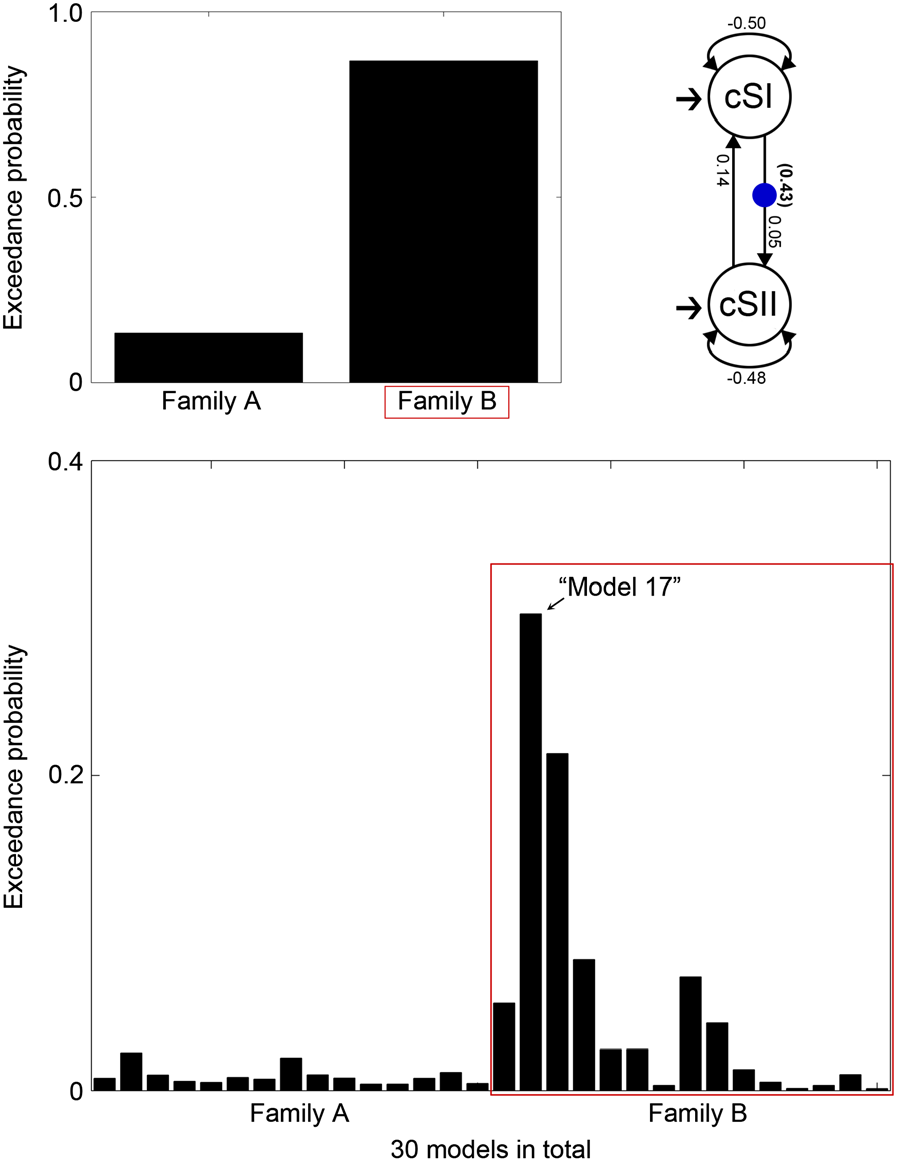
**DCM results for intra-hemispheric effective connectivity.** Family B (parallel processing, exceedance probability of 86.72%) was preferred to family A (serial processing, exceedance probability of 13.28%) (top left). The best single model (model 17) with an exceedance probability of 30.27% among 15 models in family B was selected (bottom). The best single model contained a modulation in the connection from cSI to cSII, implying a serial pathway. Numbers along the intrinsic connections indicate the mean parameters of intrinsic connectivity, and numbers in parentheses indicate the mean parameter of modulation from cSI to cSII across participants and sessions of the best single model (top right). The increase in the activity of cSII corresponds to 43% of the activity of cSI by pressure stimulation (0.43 in the parentheses from cSI to cSII).

The DCM analysis for intra-hemispheric effective connectivity revealed that family B (parallel processing) was preferred to family A (serial processing). BMS resulted in exceedance probabilities for family A and B of 13.28% and 86.72%, respectively (averaged across sessions). Among 15 single models in family B, the best model showed an exceedance probability of 30.27% (averaged across sessions). This model contained one modulation in a forward connection from cSI to cSII (a serial pathway: cSI → cSII). We confirmed the statistical significance of the modulatory parameters from cSI to cSII (*t*-test, *p* < 0.0005) across participants and sessions using the best single model (Figure 4).

**Figure 5:**
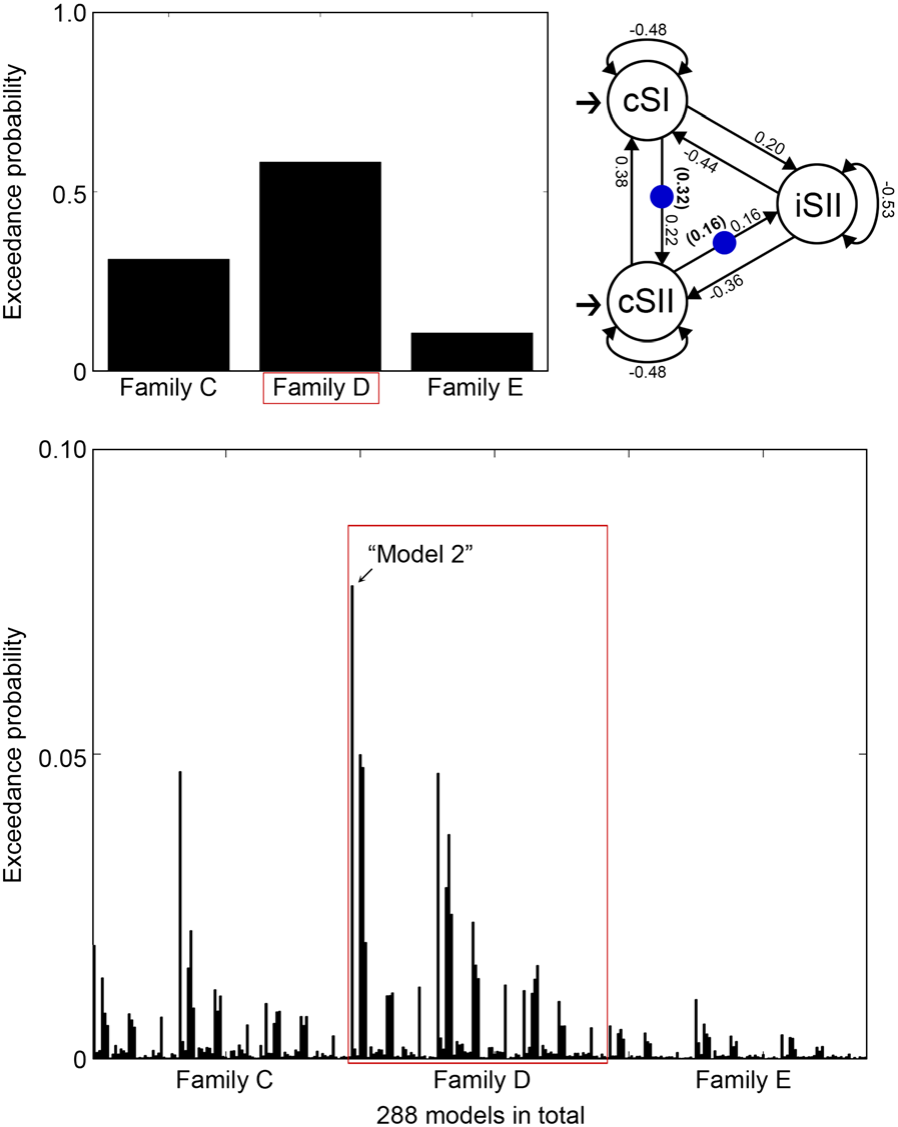
**DCM results for inter-hemispheric effective connectivity.** Family D (SII-level information flow, exceedance probability of 58.30%) was the most preferred model family, compared with family C (exceedance probability of 31.17%) and family E (exceedance probability of 10.53%) (top left). The best single model (model 2) with an exceedance probability of 7.77% among 96 models in family D was selected (bottom). The best single model contained two modulations in the connections from cSI to cSII (the serial pathway shown in intra-hemispheric effective connectivity) and from cSII to iSII. Numbers along the intrinsic connections indicate the mean parameters of intrinsic connectivity and numbers in parentheses indicate the mean parameters of modulations from cSI to cSII and from cSII to iSII across participants and sessions of the best single model (top right). The increase in the activity of cSII corresponds to 32% of the activity of cSI (0.32 in the parentheses from cSI to cSII) and the increase in the activity of iSII corresponds to 16% of the activity of cSII (0.16 in the parentheses from cSII to iSII) by pressure stimulation.

DCM analysis for inter-hemispheric effective connectivity revealed that family D (cSII → iSII) was the most preferred model family. BMS resulted in exceedance probabilities for family C, D, and E of 31.17%, 58.30%, and 10.53%, respectively (averaged across sessions). Among 96 single models in family D, we found the best model with an exceedance probability of 7.77% (averaged across sessions). This model contained two modulations, one in a forward connection from cSI to cSII (a serial pathway: cSI → cSII) and the other in a forward connection from cSII to iSII (a serial pathway: cSII → iSII). We tested the statistical significances of the modulatory parameters in the best single model from cSI to cSII (*t*-test, *p* < 0.0005) and those from cSII to iSII (*t*-test, *p* < 0.05) across participants and sessions (Figure 5).

## Discussion

### Intra-hemispheric effective connectivity

Considering the ongoing debate concerning serial and parallel processing in tactile perception, the results obtained in this study provide support for both hypotheses.

Prior evidence for the parallel processing hypothesis includes the anatomical thalamic neuronal projection from the ventroposterior thalamus (VPL) to both SI and SII in cats [38] and monkeys [39], robust activation of SII unaffected by inactivation of SI during tactile stimulation in cats [40], and simultaneous response onsets of activities in SI and SII to laser-induced nociceptive stimuli in a human magnetoencephalography (MEG) study [41]. Conversely, prior evidence for the serial processing hypothesis includes the anatomical serial connections of neurons from SI to SII in monkeys [11,12], sequential response onsets from SI to SII with temporal differences between the regional onsets of ≤ 100 ms to electrical stimuli in a human MEG study [13,42,43], and three sequential information channels from the thalamus to cSI, from cSI to cSII, and from cSII to iSII based on response onsets in cSI, cSII, and iSII during electrical stimulation in a human electroencephalography (EEG) study [14].

A review of somatosensory evoked potentials (SEPs) highlighted that SII directly received nociceptive inputs from the thalamus, whereas it mainly received tactile inputs from SI [44]. This report hinted at the possibility of the coexistence of the two hypotheses. Additionally, two human fMRI studies using DCM supported each hypothesis; one study reported that nociceptive (laser-induced heat) and non-nociceptive (electrical pulses) stimuli were directly delivered to both cSI and cSII through parallel processing [16], whereas the other study reported that vibrotactile stimuli were transmitted from cSI to cSII through serial processing [15]. Based on these previous studies, we anticipated the possibility of the coexistence of serial and parallel characteristics in tactile information processing for non-nociceptive mechanical stimuli. Our first DCM results are consistent with a study of Liang et al. [16] in terms of parallel inputs of sensory signals to cSI and cSII (family B), and also in agreement with a study of Kalberlah et al. [15] in terms of the serial transmission of sensory signals from cSI to cSII (the best model in family B contained a single modulation connection from cSI to cSII). However, our results do not perfectly connect the two previous DCM studies as Liang et al. considered models with driving inputs of electrical stimuli to the thalamus and Kalberlah et al. used vibrotactile stimuli. Thus, our study may reveal intra-hemispheric effective connectivity for another type of sensory signals from SA-I afferents characterized by: (1) the parallel processing of a bifurcated sensory input to cSI and cSII; and (2) the serial processing of sequential signal transduction from cSI to cSII. In terms of the afferent-dependency of intra-hemispheric effective connectivity based on previous and our DCM analyses, we conjecture that very high-frequency electrical stimulation (delivered by FA-II) results in parallel processing (from Liang et al. 2011 [16]), high-frequency vibrotactile stimulation (delivered by FA-I) results in serial processing (from Kalberlah et al. 2013 [15]), whereas very low-frequency vibrotactile stimulation (delivered by SA-I) recruits both processing types (from our study).

### Inter-hemispheric effective connectivity

Anatomical studies in cats [45] and monkeys [46] have shown the presence of callosal projection neurons in SI and SII and postulated inter-hemispheric transfer of somatosensory information for body representation. An animal study in monkeys revealed denser callosal connections from BA 3b to 2 implying an inter-hemispheric pathway originating from cSI [47]. A human MEG study suggested almost simultaneous delivery of sensory signals of electric stimuli to cSII and iSII in a latency ≤ 4 ms [48]. However, how tactile information is delivered from one side to the other remains controversial. It has been reported that SII has sensory neurons with bilateral receptive fields and dense callosal fibers [49]. Thus, a number of studies have suggested that the corpus callosum supports bilateral receptive fields in SII neurons for inter-hemispheric tactile information transfer. Animal studies have demonstrated decreases in the proportion of SII neurons with bilateral receptive fields in callosotomized cats with normal cats [50,51]. Human fMRI studies have demonstrated involvement of SII and the posterior parietal cortex (PPC) in inter-hemispheric tactile information transfer, by showing absence of activation responding to tactile stimuli in the ipsilateral SII and PPC in fully or partially callosotomized patients [52].

Based on these studies, we derived two hypotheses of inter-hemispheric pathways from cSI to iSII (originating from cSI) or from cSII to iSII (within SII). A recent SEP study demonstrated time-varying source connectivity during electrical stimulation such that tactile information flowed from cSI to iSII followed by information flow from cSII to iSII with a short latency of approximately 15 ms [14]. Consequently, we advanced another hypothesis of an inter-hemispheric pathway combining the other two hypotheses, both from cSI to iSII and from cSII to iSII. Among the three hypotheses, our DCM analysis results support the second hypothesis representing an SII-level inter-hemispheric pathway from cSII to iSII (family D). Additionally, the best model in family D supported serial processing as shown in the first DCM results.

The SII-level pathway for tactile information delivery we found may be attributed to the roles of SII for high-level tactile perception. Animal studies in monkeys reported the roles of SII for tactile discrimination and learning by investigating impaired task performance of monkeys after removal of the bilateral SII [53-55]. A recent human MEG-fMRI study implicated a callosal interconnection of the bilateral SII in bimanual tactile exploration of objects, revealing a significant relationship between task performance and inter-hemispheric inhibition within SII for encoding or comparing spatial features of objects [49]. Another human fMRI study suggested more complex bilateral receptive fields in SII than in SI for perceiving higher order features of tactile stimuli based on the investigation of distinct bilateral activation in SII to mechanical stimuli on fingers [56]. In addition, the role of the bilateral interaction within SII was explained by sensory-motor integration for precise control of movement executions because of multiple reciprocal connections between SII and other cortical regions (e.g. motor areas) [51].

Based on our results of two DCM analyses, we suggest that tactile information delivered through SA-I afferents by pressure stimulation is processed not only in parallel with a bifurcated input to both cSI and cSII, but also in serial with sequential sensory transmission from cSI to cSII intra-hemispherically and from cSII to iSII inter-hemispherically. These sensory information pathways are consistent with previous results that include sensory projections from thalamus to both cSI and cSII, hierarchical sensory information processing from cSI to cSII, and bilateral interactions by bilateral receptive fields within SII. Our results of inter-hemispheric connectivity underline this bilateral interaction of SII for tactile information processing by showing that pressure stimulation information from cSI more likely flows through cSII-iSII connections, not directly to iSII. Therefore, our intra- and inter-hemispheric effective connectivity model can be considered as an integrated model, which combined the three architectural factors explained above, can be used to understand low- and high-level tactile information processing of pressure stimulation.

### Limitations and future work

In our investigation of hierarchical somatosensory networks, we ruled out any temporal variation because the state equation in DCM did not consider inter-regional conduction delays [26]. We explained here only inter-regional causal activities implying information flow, not any timing issue, e.g. activation onset in cSI, cSI, and iSII. Thus, further studies are needed to investigate temporal characteristics to corroborate our effective connectivity models in view of temporal information flow during pressure stimulation.

In addition, our DCM analysis needs to be extended to other mechanical stimuli (flutter or high-frequency vibration) from FA afferents, electrical stimuli, and even nociceptive stimuli to evaluate the modality-specific consistency of our intra- and inter-hemispheric effective connectivity models. Until now, it has been reported that mechanical stimuli from FA afferents were processed in serial [15] and nociceptive stimuli were processed in parallel [16,41,57,58]. However, both serial [13,14,59] and parallel [16,39] processing modes were reported in separate studies for electrical stimulation. Our study suggests that mechanical stimuli, limited to signals from SA-I afferents (pressure stimuli), were processed both in serial and parallel. Hence, further evaluation of our models is required with other sensory stimuli from different afferents.

In terms of higher-level tactile processing, a recent neuroimaging study reported the existence of dual somatosensory pathways for the perception of texture (from SI to SII) and location (directly to SII) [60]. It therefore would be interesting to use such high-level tactile stimuli (e.g. texture or location) in addition to low-level tactile stimuli (static indentation as done here) to validate the effectiveness of our models for information processing of low-level tactile perception in the context of higher-level tasks.

Finally, causal relationships between cortical regions estimated from the fMRI data should also be examined using other assessment methods, in particular model-free methods for connectivity such as the information-theoretic transfer entropy method [61,62] to justify model-free measures of information flow.

## Conclusions

In the present study, we investigated somatosensory networks based on effective connectivity for information processing of tactile signals from SA-I afferents. Our first DCM analysis revealed that tactile signals were processed through intra-hemispheric effective connectivity characterized by parallel (sensory inputs to cSI and cSII) and serial (signal transmission from cSI to cSII) pathways, supporting *both* serial and parallel processing of tactile information. Our second DCM analysis revealed that tactile signals were transmitted serially from cSI, through cSII, to iSII over inter-hemispheric connections. Consequently, we postulate a tactile information pathway of pressure stimuli in cSI, cSII, and iSII with three components: (1) parallel processing with a bifurcated input to both cSI and cSII; (2) a serial pathway from cSI to cSII; and (3) an SII-level pathway from cSII to iSII.

## Competing interests

The authors declare that they have no competing interests.

## Authors’ contributions

YC participated in the study design, carried out the experiments and data analysis, performed the evaluation of results, and drafted the manuscript. SH participated in the study design and carried out the experiments. HK and SC provided the stimulation device for the experiments and helped perform the experiments. JP participated in the study design and helped to MRI data collection. CW conceived of the study, participated in the evaluation of results, and helped draft the manuscript. SK conceived of the study, participated in the study design and its coordination, and helped draft the manuscript. CW and SK gave final approval of the version to be published. All authors read and approved the final manuscript.

## References

[B1] Delmas P Hao J Rodat-Despoix L Molecular mechanisms of mechanotransduction in mammalian sensory neurons Nat Rev Neurosci 2011 15 3 139 153 10.1038/nrn2993 21304548

[B2] Johansson RS Flanagan JR Coding and use of tactile signals from the fingertips in object manipulation tasks Nat Rev Neurosci 2009 15 5 345 359 10.1038/nrn2621 19352402

[B3] ten Donkelaar HJ Keyser A van Domburg P The somatosensory system Clinical Neuroanatomy: Brain Circuitry and Its Disorders 2011 1 Berlin and Heidelberg: Springer-Verlag 133 209

[B4] Goodwin AW Wheat HE Sensory signals in neural populations underlying tactile perception and manipulation Annu Rev Neurosci 2004 15 53 77 10.1146/annurev.neuro.26.041002.131032 15217326

[B5] Johansson RS Dynamic use of tactile afferent signals in control of dexterous manipulation Sensorimotor Control of Movement and Posture. Volume 508 2002 New York: Springer US 397 410 10.1007/978-1-4615-0713-0_45 12171136

[B6] Maricich SM Morrison KM Mathes EL Brewer BM Rodents rely on Merkel cells for texture discrimination tasks J Neurosci 2012 15 10 3296 3300 10.1523/JNEUROSCI.5307-11.2012 22399751 PMC3306053

[B7] Dodson MJ Goodwin AW Browning AS Gehring HM Peripheral neural mechanisms determining the orientation of cylinders grasped by the digits J Neurosci 1998 15 1 521 530 9412528 10.1523/JNEUROSCI.18-01-00521.1998 PMC6793383

[B8] Bisley JW Goodwin AW Wheat HE Slowly adapting type I afferents from the sides and end of the finger respond to stimuli on the center of the fingerpad J Neurophysiol 2000 15 1 57 64 10899183 10.1152/jn.2000.84.1.57

[B9] Wheat HE Goodwin AW Browning AS Tactile resolution: peripheral neural mechanisms underlying the human capacity to determine positions of objects contacting the fingerpad J Neurosci 1995 15 8 5582 5595 7643203 10.1523/JNEUROSCI.15-08-05582.1995 PMC6577620

[B10] Friedman DP Murray EA O'Neill JB Mishkin M Cortical connections of the somatosensory fields of the lateral sulcus of macaques: evidence for a corticolimbic pathway for touch J Comp Neurol 1986 15 3 323 347 10.1002/cne.902520304 3793980

[B11] Burton H Fabri M Alloway K Cortical areas within the lateral sulcus connected to cutaneous representations in areas 3b and 1: a revised interpretation of the second somatosensory area in macaque monkeys J Comp Neurol 1995 15 4 539 562 10.1002/cne.903550405 7636030

[B12] Vogt BA Pandya DN Cortico-cortical connections of somatic sensory cortex (areas 3, 1 and 2) in the rhesus monkey J Comp Neurol 1978 15 2 179 191 10.1002/cne.901770202 413844

[B13] Inui K Wang X Tamura Y Kaneoke Y Kakigi R Serial processing in the human somatosensory system Cereb Cortex 2004 15 8 851 857 10.1093/cercor/bhh043 15054058

[B14] Hu L Zhang ZG Hu Y A time-varying source connectivity approach to reveal human somatosensory information processing Neuroimage 2012 15 1 217 228 10.1016/j.neuroimage.2012.03.094 22580382

[B15] Kalberlah C Villringer A Pleger B Dynamic causal modeling suggests serial processing of tactile vibratory stimuli in the human somatosensory cortex-An fMRI study Neuroimage 2013 15 164 171 23435215 10.1016/j.neuroimage.2013.02.018

[B16] Liang M Mouraux A Iannetti GD Parallel processing of nociceptive and non-nociceptive somatosensory information in the human primary and secondary somatosensory cortices: evidence from dynamic causal modeling of functional magnetic resonance imaging data J Neurosci 2011 15 24 8976 8985 10.1523/JNEUROSCI.6207-10.2011 21677181 PMC6622932

[B17] Karhu J Tesche CD Simultaneous early processing of sensory input in human primary (SI) and secondary (SII) somatosensory cortices J Neurophysiol 1999 15 5 2017 2025 10322043 10.1152/jn.1999.81.5.2017

[B18] Kim HS Choi MH Kim HJ Hong SP Park JY Jun JH Yi JH Chung YG Kim SP Park JR Lim DW Chung SC Development of a simple pressure and heat stimulator for intra- and interdigit functional magnetic resonance imaging Behav Res Methods 2013 in press 10.3758/s13428-013-0371-9 23861087

[B19] Tzourio-Mazoyer N Landeau B Papathanassiou D Crivello F Etard O Delcroix N Mazoyer B Joliot M Automated anatomical labeling of activations in SPM using a macroscopic anatomical parcellation of the MNI MRI single-subject brain Neuroimage 2002 15 1 273 289 10.1006/nimg.2001.0978 11771995

[B20] Eickhoff SB Stephan KE Mohlberg H Grefkes C Fink GR Amunts K Zilles K A new SPM toolbox for combining probabilistic cytoarchitectonic maps and functional imaging data Neuroimage 2005 15 4 1325 1335 10.1016/j.neuroimage.2004.12.034 15850749

[B21] Geyer S Schormann T Mohlberg H Zilles K Areas 3a, 3b, and 1 of human primary somatosensory cortex. Part 2. Spatial normalization to standard anatomical space Neuroimage 2000 15 6 Pt 1 684 696 10860796 10.1006/nimg.2000.0548

[B22] Geyer S Schleicher A Zilles K Areas 3a, 3b, and 1 of human primary somatosensory cortex Neuroimage 1999 15 1 63 83 10.1006/nimg.1999.0440 10385582

[B23] Eickhoff SB Schleicher A Zilles K Amunts K The human parietal operculum, I. Cytoarchitectonic mapping of subdivisions Cereb Cortex 2006 15 2 254 267 15888607 10.1093/cercor/bhi105

[B24] Eickhoff SB Amunts K Mohlberg H Zilles K The human parietal operculum, II. Stereotaxic maps and correlation with functional imaging results Cereb Cortex 2006 15 2 268 279 15888606 10.1093/cercor/bhi106

[B25] Friston KJ Harrison L Penny W Dynamic causal modelling Neuroimage 2003 15 4 1273 1302 10.1016/S1053-8119(03)00202-7 12948688

[B26] Stephan KE Penny WD Moran RJ den Ouden HE Daunizeau J Friston KJ Ten simple rules for dynamic causal modeling Neuroimage 2010 15 4 3099 3109 10.1016/j.neuroimage.2009.11.015 19914382 PMC2825373

[B27] Penny WD Stephan KE Daunizeau J Rosa MJ Friston KJ Schofield TM Leff AP Comparing families of dynamic causal models PLoS Comput Biol 2010 15 3 e1000709 10.1371/journal.pcbi.1000709 20300649 PMC2837394

[B28] Goebel R Roebroeck A Kim DS Formisano E Investigating directed cortical interactions in time-resolved fMRI data using vector autoregressive modeling and Granger causality mapping Magn Reson Imaging 2003 15 10 1251 1261 10.1016/j.mri.2003.08.026 14725933

[B29] Buchel C Friston KJ Modulation of connectivity in visual pathways by attention: cortical interactions evaluated with structural equation modelling and fMRI Cereb Cortex 1997 15 8 768 778 10.1093/cercor/7.8.768 9408041

[B30] Friston K Causal modelling and brain connectivity in functional magnetic resonance imaging PLoS Biol 2009 15 2 e33 10.1371/journal.pbio.1000033 19226186 PMC2642881

[B31] Friston K Moran R Seth AK Analysing connectivity with Granger causality and dynamic causal modelling Curr Opin Neurobiol 2013 15 2 172 178 10.1016/j.conb.2012.11.010 23265964 PMC3925802

[B32] Friedman DP Jones EG Burton H Representation pattern in the second somatic sensory area of the monkey cerebral cortex J Comp Neurol 1980 15 1 21 41 10.1002/cne.901920103 7410612

[B33] Burton H Carlson M Second somatic sensory cortical area (SII) in a prosimian primate, Galago crassicaudatus J Comp Neurol 1986 15 2 200 220 10.1002/cne.902470206 3722439

[B34] Manzoni T Barbaresi P Conti F Fabri M The callosal connections of the primary somatosensory cortex and the neural bases of midline fusion Exp Brain Res 1989 15 2 251 266 2670598 10.1007/BF00247886

[B35] Manzoni T Conti F Fabri M Callosal projections from area SII to SI in monkeys: anatomical organization and comparison with association projections J Comp Neurol 1986 15 2 245 263 10.1002/cne.902520208 3782508

[B36] Stephan KE Penny WD Daunizeau J Moran RJ Friston KJ Bayesian model selection for group studies Neuroimage 2009 15 4 1004 1017 10.1016/j.neuroimage.2009.03.025 19306932 PMC2703732

[B37] Demain S Metcalf CD Merrett GV Zheng D Cunningham S A narrative review on haptic devices: relating the physiology and psychophysical properties of the hand to devices for rehabilitation in central nervous system disorders Disabil Rehabil Assist Technol 2013 15 3 181 189 10.3109/17483107.2012.697532 22794937

[B38] Spreafico R Hayes NL Rustioni A Thalamic projections to the primary and secondary somatosensory cortices in cat: single and double retrograde tracer studies J Comp Neurol 1981 15 1 67 90 10.1002/cne.902030107 6273459

[B39] Zhang HQ Murray GM Coleman GT Turman AB Zhang SP Rowe MJ Functional characteristics of the parallel SI- and SII-projecting neurons of the thalamic ventral posterior nucleus in the marmoset J Neurophysiol 2001 15 5 1805 1822 11352998 10.1152/jn.2001.85.5.1805

[B40] Murray GM Zhang HQ Kaye AN Sinnadurai T Campbell DH Rowe MJ Parallel processing in rabbit first (SI) and second (SII) somatosensory cortical areas: effects of reversible inactivation by cooling of SI on responses in SII J Neurophysiol 1992 15 3 703 710 1432043 10.1152/jn.1992.68.3.703

[B41] Ploner M Schmitz F Freund HJ Schnitzler A Parallel activation of primary and secondary somatosensory cortices in human pain processing J Neurophysiol 1999 15 6 3100 3104 10368426 10.1152/jn.1999.81.6.3100

[B42] Mima T Nagamine T Nakamura K Shibasaki H Attention modulates both primary and second somatosensory cortical activities in humans: a magnetoencephalographic study J Neurophysiol 1998 15 4 2215 2221 9772274 10.1152/jn.1998.80.4.2215

[B43] Schnitzler A Volkmann J Enck P Frieling T Witte OW Freund HJ Different cortical organization of visceral and somatic sensation in humans Eur J Neurosci 1999 15 1 305 315 10.1046/j.1460-9568.1999.00429.x 9987033

[B44] Cruccu G Aminoff MJ Curio G Guerit JM Kakigi R Mauguiere F Rossini PM Treede RD Garcia-Larrea L Recommendations for the clinical use of somatosensory-evoked potentials Clin Neurophysiol 2008 15 8 1705 1719 10.1016/j.clinph.2008.03.016 18486546

[B45] Caminiti R Innocenti GM Manzoni T The anatomical substrate of callosal messages from SI and SII in the cat Exp Brain Res 1979 15 2 295 314 86455 10.1007/BF00236617

[B46] Manzoni T Barbaresi P Conti F Callosal mechanism for the interhemispheric transfer of hand somatosensory information in the monkey Behav Brain Res 1984 15 2 155 170 10.1016/0166-4328(84)90138-4 6704235

[B47] Killackey HP Gould HJ 3rd Cusick CG Pons TP Kaas JH The relation of corpus callosum connections to architectonic fields and body surface maps in sensorimotor cortex of new and old world monkeys J Comp Neurol 1983 15 4 384 419 10.1002/cne.902190403 6643713

[B48] Wegner K Forss N Salenius S Characteristics of the human contra- versus ipsilateral SII cortex Clin Neurophysiol 2000 15 5 894 900 10.1016/S1388-2457(99)00319-3 10802461

[B49] Jung P Klein JC Wibral M Hoechstetter K Bliem B Lu MK Wahl M Ziemann U Spatiotemporal dynamics of bimanual integration in human somatosensory cortex and their relevance to bimanual object manipulation J Neurosci 2012 15 16 5667 5677 10.1523/JNEUROSCI.5957-11.2012 22514328 PMC6703488

[B50] Petit D Lepore F Picard N Guillemot JP Bilateral receptive fields in cortical area SII: contribution of the corpus callosum and other interhemispheric commissures Somatosens Mot Res 1990 15 2 97 112 10.3109/08990229009144701 2378194

[B51] Picard N Lepore F Ptito M Guillemot JP Bilateral interaction in the second somatosensory area (SII) of the cat and contribution of the corpus callosum Brain Res 1990 15 1–2 97 104 2085764 10.1016/0006-8993(90)90013-2

[B52] Fabri M Polonara G Del Pesce M Quattrini A Salvolini U Manzoni T Posterior corpus callosum and interhemispheric transfer of somatosensory information: an fMRI and neuropsychological study of a partially callosotomized patient J Cogn Neurosci 2001 15 8 1071 1079 10.1162/089892901753294365 11784445

[B53] Ridley RM Ettlinger G Impaired tactile learning and retention after removals of the second somatic sensory projection cortex (SII) in the monkey Brain Res 1976 15 3 656 660 10.1016/0006-8993(76)90048-2 819106

[B54] Ridley RM Ettlinger G Further evidence of impaired tactile learning after removals of the second somatic sensory projection cortex (SII) in the monkey Exp Brain Res 1978 15 4 475 488 95960 10.1007/BF00239806

[B55] Murray EA Mishkin M Relative contributions of SII and area 5 to tactile discrimination in monkeys Behav Brain Res 1984 15 1 67 83 10.1016/0166-4328(84)90009-3 6696789

[B56] Blatow M Nennig E Durst A Sartor K Stippich C fMRI reflects functional connectivity of human somatosensory cortex Neuroimage 2007 15 3 927 936 10.1016/j.neuroimage.2007.05.038 17629500

[B57] Frot M Mauguiere F Magnin M Garcia-Larrea L Parallel processing of nociceptive A-delta inputs in SII and midcingulate cortex in humans J Neurosci 2008 15 4 944 952 10.1523/JNEUROSCI.2934-07.2008 18216202 PMC6670999

[B58] Knecht S Kunesch E Schnitzler A Parallel and serial processing of haptic information in man: effects of parietal lesions on sensorimotor hand function Neuropsychologia 1996 15 7 669 687 10.1016/0028-3932(95)00148-4 8783219

[B59] Forss N Jousmaki V Hari R Interaction between afferent input from fingers in human somatosensory cortex Brain Res 1995 15 1–2 68 76 7583255 10.1016/0006-8993(95)00424-o

[B60] Sathian K Lacey S Stilla R Gibson GO Deshpande G Hu X Laconte S Glielmi C Dual pathways for haptic and visual perception of spatial and texture information Neuroimage 2011 15 2 462 475 10.1016/j.neuroimage.2011.05.001 21575727 PMC3128427

[B61] Lizier JT Heinzle J Horstmann A Haynes JD Prokopenko M Multivariate information-theoretic measures reveal directed information structure and task relevant changes in fMRI connectivity J Comput Neurosci 2011 15 1 85 107 10.1007/s10827-010-0271-2 20799057

[B62] Schreiber T Measuring information transfer Phys Rev Lett 2000 15 2 461 464 10.1103/PhysRevLett.85.461 10991308

